# Plasma p‐tau217 for Alzheimer's disease diagnosis: a memory clinic implementation approach

**DOI:** 10.1002/dad2.70286

**Published:** 2026-02-24

**Authors:** Giorgia Brodini, Fausto Roveta, Alberto Mario Chiarandon, Silvia Boschi, Lucrezia Bonino, Elisa Maria Piella, Aurora Cermelli, Selene Limoncelli, Giulia Gioiello, Michela Zotta, Adriana Lesca, Giulio Mengozzi, Silvia Morbelli, Innocenzo Rainero, Elisa Rubino

**Affiliations:** ^1^ Department of Neuroscience “Rita Levi‐Montalcini” University of Turin Turin Italy; ^2^ Center for Cognitive Disorders and Dementias Department of Neuroscience and Mental Health AOU Città della Salute e della Scienza di Torino Turin Italy; ^3^ Department of Laboratory Medicine Clinical Biochemistry laboratory “Baldi e Riberi” A.O.U. Città della Salute e della Scienza di Torino Turin Italy; ^4^ Nuclear Medicine Unit AOU Città della Salute e della Scienza di Torino Turin Italy; ^5^ Department of Medical Sciences University of Turin Turin Italy

**Keywords:** Alzheimer's disease, FDG‐PET, plasma biomarkers, p‐tau217, real‐world study

## Abstract

**INTRODUCTION:**

Plasma phosphorylated tau at threonine 217 (p‐tau217) has shown excellent diagnostic performance for Alzheimer's disease (AD), yet real‐world validation and implementation pathways remain limited. This study assessed its clinical applicability and proposed a practical workflow for memory clinics.

**METHODS:**

Plasma p‐tau217, p‐tau181, and neurofilament light chain (NfL) were quantified in consecutive patients referred for cognitive assessment. Cerebrospinal fluid (CSF) amyloid beta (Aβ) 42/40 defined amyloid (A) status, and final etiological diagnoses were reached through multidisciplinary consensus integrating clinical, neuropsychological, and metabolic imaging data.

**RESULTS:**

Among 163 patients (mean age 69.3 ± 7.4 years, 52% female), plasma p‐tau217 distinguished A+ from A− individuals (area under the curve [AUC] 0.90, 81% sensitivity, 91% specificity). AD patients showed the highest p‐tau217 levels, while non‐AD neurodegenerative disorders exhibited elevated NfL (*p* < 0.001). A dual cut‐off strategy with 95% sensitivity and specificity could have avoided 65% of lumbar punctures.

**DISCUSSION:**

Plasma p‐tau217 demonstrated robust clinical validity and supports structured integration into routine diagnostic pathways.

## Highlights

- Plasma p‐tau217 was evaluated in a real‐world unselected memory‐clinic population.
- Plasma p‐tau217 accurately identified CSF amyloid pathology.
- A dual 95% sensitivity/specificity cut‐off strategy could have reduced lumbar punctures by 65%.
- P‐tau217 supported AD diagnosis, while NfL indicated non‐AD neurodegeneration.
- A biomarker‐driven workflow was defined for clinical implementation in memory clinics.

## 1 INTRODUCTION

The advent of novel disease‐modifying therapies for Alzheimer's disease (AD) has highlighted the critical need for an accurate and timely diagnosis, particularly during the prodromal stages.^1^ Relying exclusively on clinical features remains insufficient to distinguish AD from other forms of dementia, even for experienced clinicians, thereby highlighting the pivotal role of biomarkers in supporting diagnostic precision.^2^, ^3^

An ideal biomarker for AD should (i) capture the core pathological hallmarks of the disease – amyloid beta (Aβ) deposition and phosphorylated tau (p‑tau) accumulation being validated against neuropathologically confirmed cases; (ii) enable detection at the earliest disease stages, differentiate AD from other dementias; and (iii) be reliable, minimally invasive, cost‑effective, and readily applicable in routine clinical practice.^4^

Currently, AD diagnosis is supported by cerebrospinal fluid (CSF) biomarkers – including the Aβ42/40 ratio, total tau (t‐tau), and phosphorylated tau 181 (p‐tau181) – and by positron emission tomography (PET) imaging with [^18^F]‐fluorodeoxyglucose (FDG), amyloid, or tau tracers.^3^ However, both approaches have limitations. CSF biomarker assessment requires lumbar puncture, a relatively uncomfortable procedure that may be contraindicated in certain patients, such as those receiving anticoagulant therapy. In parallel, FDG and amyloid PET imaging, while informative, remain limited by restricted availability in several regions, higher costs compared to fluid biomarkers, and dependence on specialized equipment and operator expertise.^5^, ^6^ Moreover, tau PET imaging is not yet commercially available in Europe, further limiting its applicability in routine clinical practice.

Blood‐based biomarkers are emerging as promising tools to overcome many of these limitations.^7^ Several plasma biomarkers have been investigated,^8^ particularly Aβ and p‐tau isoforms, which align with the amyloid and tau hypotheses of AD pathogenesis.^9^ By contrast, neurofilament light chain (NfL) is regarded as a sensitive indicator of axonal damage across neurodegenerative conditions, lacking, however, specificity and thresholds.^10^, ^11^ The plasma Aβ42/40 ratio demonstrates a moderate correlation with cerebral amyloid plaque burden and can contribute to AD dementia risk prediction; however, it remains a less robust prognostic marker compared to its CSF counterpart.^12^ Among p‐tau isoforms, p‐tau217 has shown superior diagnostic accuracy, surpassing both plasma p‐tau181 and Aβ‐based markers in its correlation with AD pathology.^13^, ^14^, ^15^, ^16^ Plasma p‐tau181 shows a strong correlation with cerebral tau deposition, comparable to that observed with CSF p‐tau181 as well as tau and amyloid PET uptake.^17^ Notably, plasma p‐tau217 levels increase significantly in AD and seem to be a stronger predictor than plasma p‐tau181 since it more closely mirrors amyloid and tau PET studies.^17^ Moreover, plasma p‐tau217 appears to distinguish the AD continuum from non‐AD neurodegenerative diseases with greater accuracy than p‐tau181.^16^, ^17^ Different studies have also been focused on combinations of blood biomarkers to increase accuracy, with some contradictory outcomes.^18^, ^19^

Early studies using conventional ELISA produced conflicting plasma Aβ results, largely due to limited assay sensitivity and lack of standardized sample handling.^20^ Subsequently, advanced methods such as immunoprecipitation–mass spectrometry, single molecule array (Simoa), and fully automated chemiluminescent immunoassays were developed to measure plasma AD biomarkers, each differing in analytical sensitivity, cost, technical requirements, and accessibility.^21^ Fully automated platforms, such as Lumipulse, provide standardized, high‐throughput measurement of blood biomarkers that does not require highly specialized personnel, creating a practical opportunity for their integration into routine clinical workflows.^14^, ^22^

Although several studies have highlighted the potential of plasma p‐tau217, most have been conducted in highly selected research cohorts, typically AD‐enriched and using cognitively unimpaired individuals as controls.^11^, ^13^, ^17^ This may limit the generalizability of findings to routine clinical practice. To date, only a limited number of studies have assessed plasma p‐tau217 in unselected, real‐world clinical cohort, where diagnostic uncertainty and phenotypic heterogeneity are a common presentation.^23^, ^24^, ^25^

This study aims to evaluate the utility of plasma p‐tau217, measured using the fully automated Lumipulse platform, in a consecutive cohort representative of a real‐world memory clinic population. Patients presented with a spectrum of neurodegenerative diseases and were assessed through complementary and sequential diagnostic approaches: an initial clinical and neuropsychological evaluation, followed by a biomarker‐based assessment using CSF AD biomarkers, and subsequently an integrated framework to define individual clinic‐biological profile.

## 2 MATERIALS AND METHODS

### 2.1 Population

We consecutively enrolled individuals consulting the Center for Cognitive Disorders and Dementias at the University Hospital “Città della Salute e della Scienza di Torino” of Turin, Italy, between January 2023 and March 2025. Only individuals referred for the first time to our center were considered for inclusion in this study. The cohort included patients with conditions spanning from subjective cognitive decline (SCD) to mild cognitive impairment (MCI) and dementia. Participants were classified into cognitive stages based on the Clinical Dementia Rating (CDR) scale and consensus by at least one neurologist and one neuropsychologist. SCD was defined as self‐reported cognitive decline without objective deficits, MCI as objective cognitive impairment with preserved daily functioning, and dementia as cognitive impairment accompanied by loss of functional independence.

Inclusion criteria for this study were (i) availability of plasma p‐tau217 data and (ii) completion of at least one clinical and one neuropsychological evaluation as part of the routine diagnostic workup.

Cases were excluded if plasma p‐tau217 measurement was unavailable due to technical or logistical issues, if the patient refused to participate in the study, or if the clinical or neuropsychological evaluation was not completed because of early dropout from the clinic. Demographic and other clinical information, including renal function or cardiovascular comorbidities, were collected. Global cognitive status was assessed in all participants using the Mini‐Mental State Examination (MMSE) or the Montreal Cognitive Assessment (MoCA), complemented by additional neuropsychological tests as part of the standard memory clinic evaluation. Subgroups of participants underwent additional investigations, including CSF analysis for Aβ42/40 and p‐tau181 levels and brain FDG‐PET. Only lumbar punctures performed within 3 months of blood sampling, and FDG‐PET scans acquired within 1 year of blood sampling were considered for this study. Given the opportunity to apply the AT(N) framework through CSF biomarkers and our secondary focus on differential diagnosis, FDG‐PET rather than amyloid PET was incorporated into the diagnostic flowchart. This choice also reflects the real‐world context of our cohort, as FDG‐PET remains more widely available than amyloid PET in Italy. A subgroup of participants underwent apolipoprotein E (*APOE*) genotyping. We used the Standards for Reporting of Diagnostic Accuracy (STARD) checklist when writing our report.^26^

### 2.2 Biological samples and imaging protocols

#### 2.2.1 Biological samples

CSF and blood samples were collected following previously described standardized protocols.^27^ Briefly, CSF was obtained via lumbar puncture using an atraumatic needle after overnight fasting, collected in polypropylene tubes, centrifuged within 1 h at 2000 × g for 10 min at room temperature, then aliquoted and stored at −80°C. Concentrations of Aβ42, Aβ40, p‐tau, and p‐tau181 were measured in all CSF samples using the Lumipulse G600II platform (Fujirebio Europe, Ghent, Belgium) at the Clinical Biochemistry Laboratory of our university hospital. Aβ positivity (“A”) was defined as CSF Aβ42/40 ratio < 0.068 and tau positivity (“T”) as CSF p‐tau181 > 61.0 pg/mL, according to established thresholds.^28^ Plasma was collected in EDTA tubes, kept at room temperature for up to 2 h prior to centrifugation (1700 × g, 15 min), aliquoted (500 µL), and stored at −80°C in the biobank of our Department. Plasmatic p‐tau217, p‐tau181, and NfL were measured using the Lumipulse G600II instrument with commercial kits (Fujirebio Europe, Ghent, Belgium). Plasma samples were analyzed in one analytical batch after collection and storage. DNA was extracted from whole blood, and apolipoprotein E (*APOE*) was genotyped.

**RESEARCH IN CONTEXT**

1. **Systematic review**: We reviewed recent literature focusing on plasma phosphorylated tau biomarkers, particularly p‐tau217, with emphasis on diagnostic performance across clinical, community, and biomarker‐defined cohorts, as well as studies employing automated platforms and real‐world samples. Most evidence derives from research‐enriched, amyloid‐positive populations versus healthy controls, limiting generalizability to heterogeneous memory‐clinic settings.
2. **Interpretation**: Our results confirm that plasma p‐tau217 maintains high diagnostic accuracy for detecting CSF‐defined amyloid pathology in an unselected, consecutive memory‐clinic cohort, outperforming p‐tau181 and aligning with clinical, biological, and etiological classifications. The proposed dual cut‐off strategy provides rule‐in/rule‐out thresholds and could meaningfully reduce lumbar punctures while supporting implementation pathways.
3. **Future directions**: Future studies must validate cut‐offs in independent and multicenter cohorts, assess value within disease‐modifying therapy pathways, determine health‐economic impact, and explore multimodal integration to refine differential diagnosis.

#### 2.2.2 Classification of subjects into biological A/T status and etiological diagnostic groups

Patients were classified according to the Alzheimer's Association framework using CSF biomarker profiles for the purpose of biological categorization.^29^ Based on 2024 revised criteria for the diagnosis of AD, individuals were categorized as either A− or A+ using available CSF Aβ42/Aβ40 ratio as Core 1 criteria, allowing evaluation of the diagnostic performance of plasma p‐tau217 in identifying biologically defined AD.^30^ All patients underwent a comprehensive diagnostic workup, including neurological and cognitive assessments, structural neuroimaging (MRI or CT), and additional investigations when clinically indicated, as previously described.^27^ Final etiological diagnoses were made through a multidisciplinary consensus involving neurologists, neuropsychologists, and nuclear medicine physicians, based on validated diagnostic criteria and following current European recommendations for the biomarker‐based diagnosis of neurocognitive disorders.^3^, ^31^, ^32^, ^33^, ^34^ Plasma biomarker results were unavailable at the time of definition of final etiological diagnosis. Patients with suspected behavioral variant frontotemporal dementia, non‐AD primary progressive aphasia, or atypical parkinsonian syndromes (mainly progressive supranuclear palsy and corticobasal syndrome phenotype) were classified within the probable frontotemporal lobar degeneration (FTLD) group. Individuals with A−/T− status and no clinical, cognitive, or imaging features suggestive of a neurodegenerative disorder were classified into the probable non‐neurodegenerative disease (NND) group. Patients diagnosed with other probable non‐AD neurodegenerative diseases (e.g., dementia with Lewy bodies or prion disease) were categorized as other neurodegenerative disorders (OND). Etiological diagnoses were confirmed at follow‐up visits. Cases with ongoing diagnostic uncertainty or lacking sufficient data for definitive classification were excluded from etiological subgroup analyses.

#### 2.2.3 Brain FDG‐PET

The PET images were acquired at the Nuclear Medicine Unit of the University Hospital “Città della Salute e della Scienza di Torino” according to international procedural guidelines.^35^ FDG‐PET was mainly used as a supporting criterion for non‐AD etiological diagnoses^36^, ^37^ and in cases lacking CSF‐based A/T classification for the purpose of etiological categorization. In these cases, AD diagnosis was inferred – within the context of a typical clinical syndrome and multidisciplinary evaluation – based on the presence of a characteristic posterior hypometabolic pattern, as determined by an experienced nuclear medicine physician.^38^ FDG‐PET scans were performed within an average interval of 3.8 ± 2.4 months from blood sampling.

### 2.3 Statistical analysis

Baseline differences in demographics, clinical features, cognitive performance, and biomarker levels across cognitive stages (SCD, MCI, dementia), A/T status, and etiological diagnostic groups were assessed using the Kruskal–Wallis test for continuous variables and the chi‐squared test for categorical variables. Post hoc pairwise comparisons were performed using the Dunn's test with Bonferroni correction for multiple comparisons. Spearman's correlation was used to assess the relationship between plasma and CSF biomarker levels. Logistic regression models were employed to evaluate the association between plasma biomarkers and amyloid status, with age, sex, education, presence of cardiovascular risk factors, and *APOE* status included as covariates. Receiver operating characteristic (ROC) curve analyses were used to determine the diagnostic accuracy of plasma biomarkers in distinguishing A− from A+ groups. Optimal cut‐off values were identified using the Youden index. Positive predictive value (PPV), negative predictive value (NPV), and accuracy were calculated for selected cut‐offs based on the prevalence observed in the study cohort. We further explored the application of a double cut‐off strategy for plasma p‐tau217. Specifically, we adopted an approach in which the upper cut‐off was defined to achieve 95% specificity, while the lower cut‐off was established to ensure 95% sensitivity. For this dual cut‐off approach, PPV, NPV, and accuracy were calculated only among classified cases (i.e., values below the lower cut‐off or above the upper cut‐off). As sensitivity analysis, we applied the double cut‐offs identified in the Palmqvist et al. study for comparison purposes.^23^ Statistical analyses were conducted using R (version 2025.4.5.2) and Jamovi (version 2.3.28); a significance level of *p* < 0.05 was adopted.

## 3 RESULTS

A total of 163 patients met the inclusion criteria for this study. Key demographic and clinical information, neuropsychological features, and available biomarkers information of participants by diagnostic stages (SCD, MCI, dementia) are presented in Table 1. A flow chart of participants illustrating the classification of subjects into biological and etiological diagnostic groups is presented in Figure S1.

**TABLE 1:** Demographics, cognitive features, and biomarker status of subjects by cognitive stages.

Variable	Overall (*n* = 163)	SCD (*n* = 18)	MCI (*n* = 89)	Dementia (*n* = 56)	*p* value
**Demographic**					
Age (years)	163	67.6 (10.01)	70.2 (6.86)	68.4 (7.31)	0.274
Gender, female	163	8 (44%)	39 (44%)^b^	37 (66%)^a^	**0.028**
Years of education	155	12.6 (4.53), *N* = 18	11.4 (4.30), *N* = 82	9.9 (3.73), *N* = 55	**0.045**
**Cardiovascular risk and renal function**					
0 to 1 risk factor/≥2 risk factors (%)	163	22/78	37/63	48/52	0.13
eGFR (mg/dL)	131	89.10 (12.52) *N* = 10	80.50 (17.63) *N* = 76	84.60 (17.83) *N* = 45	0.256
**Cognitive features**					
MMSE	161	28.9 (1.08)^a^ *N* = 18	26 (2.54)^b^ *N* = 88	17.6 (5.69)^c^ *N* = 55	**<0.001**
MoCA	119	27.2 (1.48)^a^ *N* = 10	19.1 (3.39)^b^ *N* = 73	12.1 (2.79)^c^ *N* = 36	**<0.001**
**Biomarkers**					
CSF Aβ42/40	120	0.089 (0.017)^a^ *N* = 5	0.061(0.024) *N* = 71	0.054 (0.022)^b^ *N* = 44	**0.010**
CSF p‐tau181 (pg/mL)	120	36.9 (6.37)^b^ *N* = 5	68.8 (45.3)^b^ *N* = 71	106 (86.6)^a^ *N* = 44	**0.008**
Plasma p‐tau217 (pg/mL)	163	0.250 (0.454)^b^	0.351 (0.320)^a^	0.596 (0.494)^a^	**0.004**
Plasma p‐tau181 (pg/mL)	139	1.11 (0.708)^b^ *N* = 17	1.50 (1.05) *N* = 74	2.03 (1.36)^a^ *N* = 48	**0.010**
Plasma NfL (pg/mL)	113	18.5 (7.53)^c^ *N* = 16	29.8 (18.5)^b^ *N* = 74	43.9 (33.8)^a^ *N* = 36	**<0.001**
*APOE* ε4 carrier	106	2 (67%)	21 (32%)	16 (42%)	0.477
PET‐FDG with AD‐like pattern	105	1 (20%)	25 (42%)	26 (63%)	**0.048**
PET‐FDG with non‐AD pattern	105	1 (20%)	21 (36%)	8 (20%)	0.20

*Note*: Numerical data are presented as the mean (standard deviation), while categorical data are expressed as the number (percentage of the total). Post hoc comparisons are reported as a > b > c.

Abbreviations: APOE, apolipoprotein E; CSF, cerebrospinal fluid; eGFR, estimated glomerular filtration rate; MCI, mild cognitive impairment; MMSE, Mini‐Mental State Examination; MoCA, Montreal Cognitive Assessment; SCD, subjective cognitive decline.

Female gender distribution differed significantly between dementia (66%) and MCI (44%) patients (*p* = 0.028). No significant differences were found among the groups in terms of age, renal function, or presence of cardiovascular risk factors. Plasma p‐tau217 levels were different across cognitive, biological, and etiological categorization (Figure S2).

### 3.1 Blood biomarkers across cognitive stages

Plasma p‐tau217 levels were elevated in both MCI (*N* = 89, 0.351 ± 0.320 pg/mL) and dementia (*N* = 56, 0.596 ± 0.494 pg/mL) compared to SCD (*N* = 18, 0.250 ± 0.454 pg/mL) (*p* = 0.004). No significant differences emerged between MCI and dementia groups (*p* = 0.088). Plasma NfL levels varied significantly across cognitive stages, with concentrations of 18.5 ± 7.53pg/mL in SCD, 29.8 ± 18.5 pg/mL in MCI, and 43.9 ± 33.8 pg/mL in dementia (*p* < 0.001).

### 3.2 Blood biomarkers across CSF A/T status

A strong inverse correlation between plasma p‐tau217 levels and CSF Aβ42/40 ratio was found (rho = −0.673, *p* < 0.001). Plasma p‐tau217 levels were higher in the A+/T+ group (*N* = 55, 0.754 ± 0.422 pg/mL) than the other groups (*p* < 0.001); a significant difference was also found between A+/T− (*N* = 20, 0.360 ± 0.294 pg/mL) and A−/T− (*N* = 41, 0.144 ± 0.120 pg/mL) participants (Table 2).

**TABLE 2:** Demographics, cognitive features, and plasma biomarkers of subjects by A/T status.

Variable	Overall (*n* = 120)	A−/T−*N* = 41	A−/T+*N* = 4	A+/T−*N* = 20	A+/T+*N* = 55	*p* value
**Demographic**						
Age (years)	120	67.3 (8.38)	73.3 (4.35)	72.4 (4.07)	69.1 (7.34)	0.076
Gender, female	120	19 (46%)	2 (50%)	11 (55%)	29 (53%)	0.920
Years of education	119	13.1 (4.45)^a^ *N* = 40	7.5 (1.73) *N* = 4	9.45 (3.49)^b^ *N* = 20	10.9 (3.88) *N* = 55	**0.004**
**Cognitive features**						
SCD/MCI/dementia (%)	120	10/68/22	0/50/50	5/50/45	0/56/44	0.062
MMSE	120	26 (3.85)^a^	24.5 (4.51)	23.4 (4.28)	21.1 (6.47)^b^	**<0.001**
MoCA	106	19.3 (5.01) *N* = 38	16 (4.90) *N* = 4	16.1 (4.59) *N* = 20	16.2 (5.11) *N* = 44	0.062
**Biomarkers**						
Plasma p‐tau217 (pg/mL)	120	0.144 (0.120)^b.b1^	0.104 (0.032)^b^	0.360 (0.294)^b.a1^	0.754 (0.422)^a^	**<0.001**
Plasma p‐tau181 (pg/mL)	96	0.93 (0.41)^b^ *N* = 31	1.67 (1.77) *N* = 4	1.28 (0.53)^b^ *N* = 16	2.57 (1.35)^a^ *N* = 45	**<0.001**
Plasma NfL (pg/mL)	71	35.3 (20.3) *N* = 10	27.4 (10.0) *N* = 3	47.9 (43.6) *N* = 10	27.3 (11.6) *N* = 34	0.292
*APOE* ε4 carrier	103	5 (14.71%)^b^ *N* = 34	0 (0%) *N* = 4	9 (50%) *N* = 18	25 (53.19%)^a^ *N* = 47	**<0.001**

Note: Numerical data are presented as the mean (standard deviation), while categorical data are expressed as the number (percentage of the total). Post‐hoc comparisons are reported as a > b > c > d.

Abbreviations: APOE, apolipoprotein E; MCI, mild cognitive impairment; MMSE, Mini‐Mental State Examination; MoCA, Montreal Cognitive Assessment; SCD, subjective cognitive decline.

**FIGURE 1:**
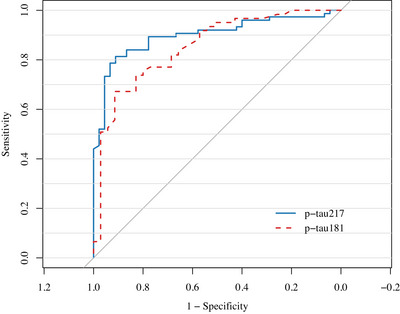
Receiver operating characteristic curves of plasma p‐tau217 and p‐tau181 predicting CSF A− versus A+. AUC, area under the curve.

ROC analysis (*N* = 120) supported the high diagnostic accuracy of plasma p‐tau217 in distinguishing CSF A+ status from A−, with an AUC of 0.90, 95% CI 0.84 to 0.96 (sensitivity: 81%, specificity: 91%), outperforming plasma p‐tau181 (AUC 0.85, 95% CI 0.77 to 0.93, sensitivity: 67%, specificity: 91%) (Figure 1).

**FIGURE 2:**
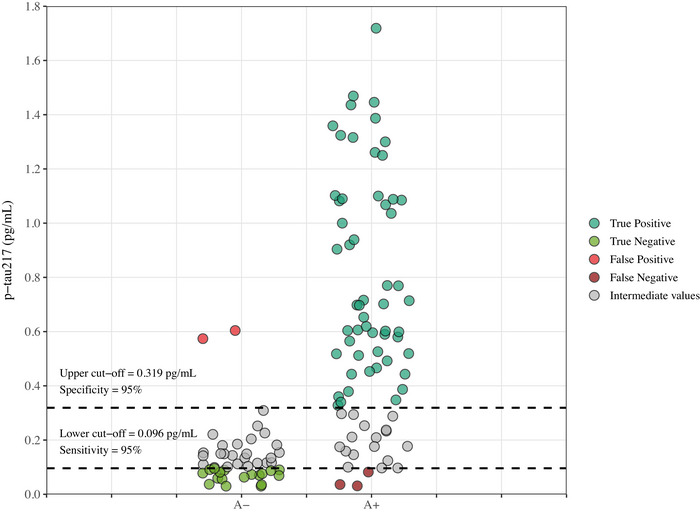
Dual cut‐off approach of p‐tau217 for CSF A− versus A+. Scatterplot shows plasma tau phosphorylated at threonine 217 (p‐tau217) concentrations according to cerebrospinal fluid amyloid status (A−/A+).

Using the AUC‐derived optimal threshold, a plasma p‐tau217 concentration of 0.230 pg/mL provided the best discrimination of CSF amyloid status in our cohort. A dual cut‐off strategy further improved clinical interpretability: values < 0.096 pg/mL ruled out AD with 95% sensitivity, while values > 0.319 pg/mL ruled in with 95% specificity. Table S1 reports the PPVs, NPVs, and accuracy associated with the identified cut‐offs. Applied to cases with available CSF data, 17.5% of patients fell below the lower cut‐off and 47.5% exceeded the upper cut‐off (Figure S3). This approach misclassified three CSF A+ cases (false negatives) and two CSF A− cases (false positives) out of 120 cases (Figure 2).

For comparison, we then applied the plasma p‐tau217 threshold values previously identified by Palmqvist et al. (0.220 pg/mL for 95% sensitivity and 0.340 pg/mL for 95% specificity) as a sensitivity analysis.^23^ The 0.220 pg/mL cut‐off ruled out AD with 81% sensitivity, while values >0.340 pg/mL ruled in AD with 96% specificity. Table S2 provides the PPVs, NPVs, and accuracy associated with these cut‐offs.

Individuals within the intermediate plasma p‐tau217 range showed heterogeneous clinical and biological profiles (Table S3). As shown in Figure S4, many exhibited CSF Aβ42/40 ratios and p‐tau181 levels clustering near established cut‐offs, while most cases displayed an A−/T− profile and were associated with non‐AD etiologies.

Logistic regression (adj. *R* ^2^ = 0.507, *p* < 0.001) confirmed plasmatic p‐tau217 as a strong independent predictor of A+ status versus A− (*B* = 10.73, *SE* = 3.26, *p* = 0.001), independent of age, sex, education, cardiovascular risk, or *APOE* status.

### 3.3 Blood biomarkers across etiological diagnostic groups

A total of 130 participants had sufficient clinical, CSF, or FDG‐PET data to allow classification into a probable etiological group: 68 fulfilled criteria for AD, 25 for FTLD, 15 for OND, and 22 for NND (Table 3). The remaining 33 patients had inconclusive or ongoing assessments and were not assigned to any group. To illustrate the integration of multimodal information in clinical practice, Figure S5 shows two representative cases with complete clinical, biomarker, and imaging data.

**TABLE 3:** Demographics, cognitive features and plasma biomarkers of subjects by etiological group.

Variable	Overall (*n* = 130)	AD *N* = 68	FTLD *N* = 25	OND *N* = 15	NND *N* = 22	*p* value
**Demographic**						
Age (years)	130	71 (6.72)	67.8 (7.20)	71.5 (5.63)	67.4 (9.51)	0.357
Gender, female	130	37 (54%)	9 (36%)	7 (47%)	13 (60%)	0.370
Years of education	125	10.5 (3.86) *N* = 68	13.3 (4.23) *N* = 22	10.8 (3.83) *N* = 13	12 (4.87) *N* = 22	0.062
**Cognitive features**						
SCD/MCI/dementia (%)	130	2/51/47	0/68/32	0/60/40	27/64/9	**<0.001**
MMSE	129	21.1 (6.12)^b^ *N* = 68	24.8 (4.89)^a^ *N* = 24	23.3 (4.98) *N* = 15	27.1 (3.06)^a^ *N* = 22	**<0.001**
MoCA	106	15.6 (4.92)^b^ *N* = 56	17.1 (4.03)^b^ *N* = 19	17.3 (3.92) *N* = 12	22.1 (4.48)^a^ *N* = 19	**<0.001**
**Biomarkers**						
CSF available	116	64 (94%)	22 (88%)	11 (73%)	19 (86%)	0.094
CSF Aβ+	116	63 (98.4%)^a^	2 (9.1%)^b^	4 (36%)^b^	2 (11%)^b^	**<0.001**
Plasma p‐tau217 (pg/mL)	130	0.712 (0.406)^a^	0.192 (0.261)^b^	0.216 (0.208)^b^	0.154 (0.168)^b^	**<0.001**
Plasma p‐tau181 (pg/mL)	106	2.34 (1.23)^a^ *N* = 55	1.20 (1.24)^b^ *N* = 19	1.36 (1.03)^b^ *N* = 14	1.04 (0.529)^b^ *N* = 18	**<0.001**
Plasma NfL (pg/mL)	81	28.8 (13.0)^b^ *N* = 40	49.6 (18.8)^a^ *N* = 17	60.8 (43.2)^a^ *N* = 10	18.4 (5.1)^b^ *N* = 14	**<0.001**
*APOE* ε4 carrier	100	31 (55.4%)^a^ *N* = 56	3 (15.8%)^b^ *N* = 19	2 (22.2%) *N* = 9	2 (12.5%)^b^ *N* = 16	**0.001**

Note: Numerical data are presented as the mean (standard deviation), while categorical data are expressed as the number (percentage of the total). Post‐hoc comparisons are reported as a > b > c > d.

Abbreviations: AD, Alzheimer's disease; APOE, apolipoprotein E; FTLD, frontotemporal lobar degeneration; OND, other neurodegenerative disorders; NND, non‐neurodegenerative disorders; MCI, mild cognitive impairment; MMSE, Mini‐Mental State Examination; MoCA, Montreal Cognitive Assessment; SCD, subjective cognitive decline.

Plasma p‐tau217 levels were significantly elevated in AD patients (0.712 ± 0.406 pg/mL) compared with those with FTLD (0.192 ± 0.261 pg/mL, *p* < 0.001), OND (0.216 ± 0.208 pg/mL, *p* < 0.001), and NND (0.154 ± 0.168 pg/mL, *p* < 0.001). Notably, 15 of the 25 FTLD (60%) patients fell within the intermediate values defined by our dual cut‐off strategy. No significant differences in plasma p‐tau217 concentrations were observed among the non‐AD subgroups.

Plasma NfL levels differed across diagnostic groups, with the highest concentrations observed in OND (60.8 ± 43.2 pg/mL) and FTLD (49.6 ± 18.8 pg/mL), both significantly exceeding values in AD (28.8 ± 13.0 pg/mL) and NND (18.4 ± 5.1 pg/mL) (*p* < 0.001). Plasma NfL values in AD patients were higher compared to NND individuals with only a borderline significance (*p* = 0.059). The optimal plasma NfL threshold to discriminate AD from non‐AD neurodegenerative disorders (OND + FTLD groups) was 35.17 pg/mL, corresponding to an AUC of 0.82 (95% CI 0.71 to 0.93), with 78% sensitivity and 80% specificity. In an exploratory analysis restricted to individuals with plasma p‐tau217 values below the rule‐in cut‐off and excluding AD cases etiologically defined, plasma NfL showed excellent discrimination between non‐AD neurodegenerative diseases and non‐neurodegenerative conditions (*AUC* = 0.94, 95% CI 0.86 to 1.00), and a threshold of approximately 27.5 pg/mL achieved high specificity (100%) and sensitivity (92%); these findings are illustrated in Figure S6.

## 4 DISCUSSION

This study confirms the role of plasma p‐tau217 as a robust and reliable blood‐based biomarker for AD, evaluated here in a real‐world, unselected cohort of consecutive patients attending our memory clinic and measured using a fully automated platform readily implementable in clinical practice. Unlike many previous investigations – often conducted in research cohorts enriched with AD cases and cognitively unimpaired controls – our study reflects the heterogeneity and diagnostic uncertainty characteristic of a real‐world neurological memory clinic, representing a critical step toward validating p‐tau217 for routine clinical use.^22^, ^25^

In line with prior studies, we observed a strong correlation between plasma p‐tau217 levels and the CSF Aβ42/40 ratio, confirming its value as a marker of underlying amyloid pathology.^15^, ^39^, ^40^ Notably, p‐tau217 discriminated individuals with CSF A+ status from A− cases with high diagnostic accuracy (*AUC* = 0.90), outperforming p‐tau181. Overall, these findings support and extend previous research by demonstrating the biomarker reliability in more heterogeneous clinical populations.^25^, ^41^

A distinctive strength of this study is the assessment of multiple plasma biomarkers across different classification frameworks: clinical‐neuropsychological (cognitive staging), biological (AT[N] framework for the AD continuum), and etiological (integrated clinical‐biological approach). This multimodal strategy enabled us to evaluate the utility of plasma biomarkers from several complementary perspectives. Within a memory clinic setting, each patient can be classified into one of the three traditional cognitive stages: SCD, MCI, or dementia. In this context, plasma NfL emerged as the most reliable biomarker for differentiating these categories. Plasma p‐tau217 and p‐tau181 also showed significant differences, particularly when comparing cognitively impaired (MCI and dementia) to cognitively unimpaired (SCD) individuals – reflecting the predominance of AD as the leading cause of cognitive decline in a typical memory clinic cohort.^25^ Applying the biological classification according to the AT(N) framework,^29^ we characterized plasma p‐tau217 and NfL values across the AD continuum. Within our memory cohort, characterized by a relative high prevalence of alternative etiologies, plasma p‐tau217 clearly distinguished A+ from A− cases, whereas NfL did not.

From an etiological perspective, and leveraging an integrated clinic‐biological approach including FDG‐PET disease‐related patterns, plasma p‐tau217 and NfL showed consistent yet complementary profiles: plasma p‐tau217 was markedly elevated in AD, whereas NfL was higher in non‐AD neurodegenerative conditions, in line with previous reports.^42^, ^43^

By applying a dual cut‐off strategy for p‐tau217, we established thresholds to better distinguish AD from non‐AD cases. Using this approach, we estimated that 65% of lumbar punctures performed to confirm AD biological diagnosis could have been avoided – an impactful proportion, aligned with recent findings.^44^ This strategy could enhance the diagnostic efficiency of memory clinics, enabling more rapid identification of patients potentially eligible for disease‐modifying treatments. Interestingly, 60% of patients etiologically classified as FTLD fell into the “gray zone” defined by this dual cut‐off approach, underscoring the need for a multimodal clinical‐biological strategy for the rational use of plasma biomarkers.

The largest study to date using the fully automated Lumipulse platform conducted by Palmqvist et al. has proposed clinically applicable plasma p‐tau217 cut‐offs, which we used as an external validation in a sensitivity analysis.^23^ In our cohort, these thresholds yielded a sensitivity of 81% and a specificity of 96%, confirming assay robustness but showing differences compared with our locally derived cut‐offs. These discrepancies likely reflect the heterogeneity of a real‐world memory clinic population with a high prevalence of non‐AD neurodegenerative disorders but may also be influenced by differences in AD prevalence, disease stage distribution, reference standards, and analytical or pre‐analytical variability across cohorts. Overall, these findings highlight the need to contextualize cut‐offs and support local validation when implementing plasma biomarkers into clinical practice.

Building on these findings and previous literature, we propose a practical workflow for the use of plasma biomarkers in a memory clinic setting. For patients with suspected cognitive neurodegenerative disease, the initial step involves clinical‐neuropsychological characterization and assignment to a cognitive stage. If further evaluation for suspected AD is indicated, p‐tau217 should be assessed using the dual cut‐off approach in case of confirmed cognitive impairment. Values below the lower cut‐off can effectively rule out AD with high accuracy, after which further investigations should be guided by clinical judgment.

**FIGURE 3:**
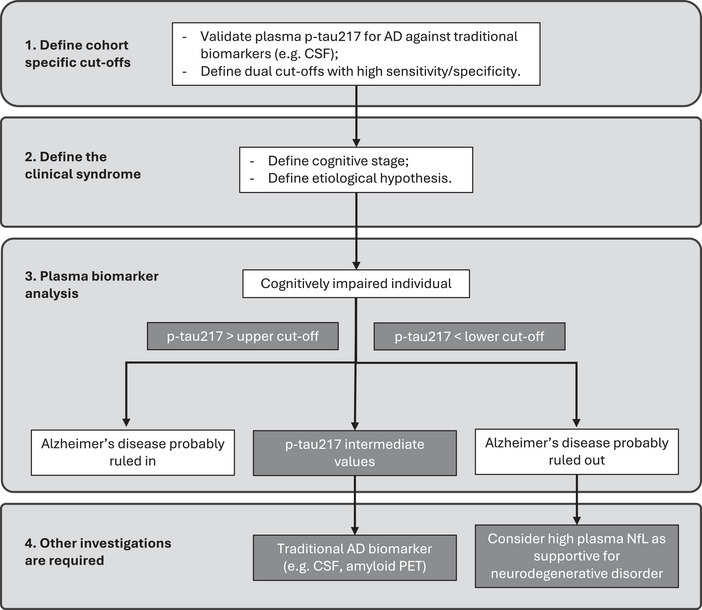
Proposed diagnostic workflow for integrating plasma biomarkers into memory clinic practice. The flow chart outlines sequential steps: establishing cohort‐specific cut‐offs, defining clinical syndrome, performing plasma biomarker analysis, and guiding further investigations, withp‐tau217 thresholds used to rule in or rule out AD and NfL supporting identification of non‐AD neurodegenerative disorders. AD, Alzheimer's disease; CSF, cerebrospinal fluid; NfL, neurofilament light chain.

Values above the upper cut‐off strongly support AD as the likely etiology in a coherent clinical context, while patients with intermediate values should undergo second‐level investigations, including CSF or PET assessment. Plasma NfL may support the hypothesis of an underlying neurodegenerative etiology. As suggested by previous studies, the combined interpretation of p‐tau217 and NfL may improve differentiation between FTLD or other neurodegenerative conditions and AD, and our results support this potential clinical utility. Figure 3 summarizes this proposed workflow for clinical practice in a memory clinic. It is important to note that each center implementing plasma biomarkers should determine its own cohort‐specific cut‐offs, as these may vary and reflect differences in referral populations.^45^ For this reason, we have included the definition of local specific as a preliminary step for the implementation of plasma biomarkers in the clinical setting of a memory clinic. Such a model could support diagnostic triage algorithms and reduce the burden on patients and healthcare systems.

Our study demonstrates the feasibility of measuring p‐tau217 using the fully automated Lumipulse platform. Nonetheless, some limitations should be acknowledged. The relatively small size of certain subgroups (e.g., OND) and the absence of an independent validation cohort warrant caution in generalizing the proposed approach. Further multicenter and longitudinal studies are needed to confirm these findings, define prognostic performance, and optimize the integration of p‐tau217 into diagnostic and therapeutic decision‐making. Additionally, incomplete availability of certain data (e.g., APOE) may have underpowered specific comparisons. FDG‐PET was not available for all patients, and amyloid PET was not included in our clinical workflow. Although amyloid PET is regarded as an in vivo gold standard for validating biomarkers of brain amyloidosis, its use in European memory clinics remains limited, largely due to reimbursement constraints. By contrast, FDG‐PET is still more widely employed and, according to European consensus recommendations for MCI and early dementia, represents the preferred option when CSF biomarkers are inconclusive, particularly to support differential diagnosis among non‐AD etiologies.^3^ The role of plasma biomarkers to support the differential diagnosis was indeed one of the aims of the present study. A further limitation is that plasma biomarkers were analyzed in a single measurement rather than prospectively, which could have reduced the analytical variability and may not fully reflect routine clinical implementation, where measurements are performed continuously. An additional limitation is that body mass index and other metabolic variables were not systematically collected, precluding assessment of the potential influence of obesity and metabolic factors on plasma biomarker levels. Finally, as diagnostic testing followed an indication‐driven approach, and despite the consecutive enrollment of participants, selection bias cannot be excluded.

In conclusion, our study demonstrates that plasma p‐tau217, measured with a fully automated and widely accessible platform, is a highly informative and clinically relevant biomarker for AD in a real‐world memory‐clinic cohort. By validating its diagnostic performance in an unselected population and introducing a dual cut‐off strategy, we confirm its accuracy and provide a practical, data‐driven framework for clinical implementation. This approach illustrates how plasma p‐tau217 can enhance diagnostic precision and reduce reliance on relatively invasive or costly procedures, supporting its integration into routine workflows and paving the way for earlier diagnosis and informed therapeutic decision‐making in the era of disease‐modifying treatments.

## AUTHOR CONTRIBUTIONS

G.B., F.R., and E.R. designed and conceptualized the study. G.B. and F.R. played a major role in the acquisition of data, analyzed and interpreted the data, and drafted the manuscript. A.M.C. analyzed and interpreted the data. S.B., L.B., E.M.P., A.C., S.L., G.G., M.Z., and A.L. had a role in the acquisition of data. G.M., S.M., and I.R. supervised the study. All authors contributed to drafting and reviewing the manuscript.

## CONFLICT OF INTEREST STATEMENT

S. Morbelli received speaker honoraria from GE healthcare, Eli Lilly, Eisai, Life Molecular Imaging, and Novartis. I. Rainero received speaker honoraria from Eli Lilly, Pfizer, and AbbVie. The remaining authors declare that they have no relevant financial interests in this manuscript. Author disclosures are available in the Supporting Information.

## CONSENT STATEMENT

The study was approved by the local Ethics Committee (protocol number 0143406/2022) and conducted in accordance with the Declaration of Helsinki and Good Clinical Practice guidelines. Written informed consent was obtained from all participants.

## Supporting information



Supporting information

Supporting information

## Data Availability

The datasets used and/or analyzed during the current study are available on request. Please contact the corresponding author.
