# Supplementary material for: Plasma p‐tau217 for Alzheimer's disease diagnosis: a memory clinic implementation approach
Source: Alzheimers Dement (Amst). 2026 Feb 24;18(1):e70286. doi: 10.1002/dad2.70286 (PMC12932973; doi:10.1002/dad2.70286)
Supplement: Supplementary file 1 — Supporting information [file DAD2-18-e70286-s002.docx]

**Supplementary Materials**

**Plasma p‑tau217 for Alzheimer’s disease diagnosis: A memory clinic implementation approach**

**Figure S1.** Flowchart for classification of subjects into cognitive, biological, and etiological diagnostic groups.

**
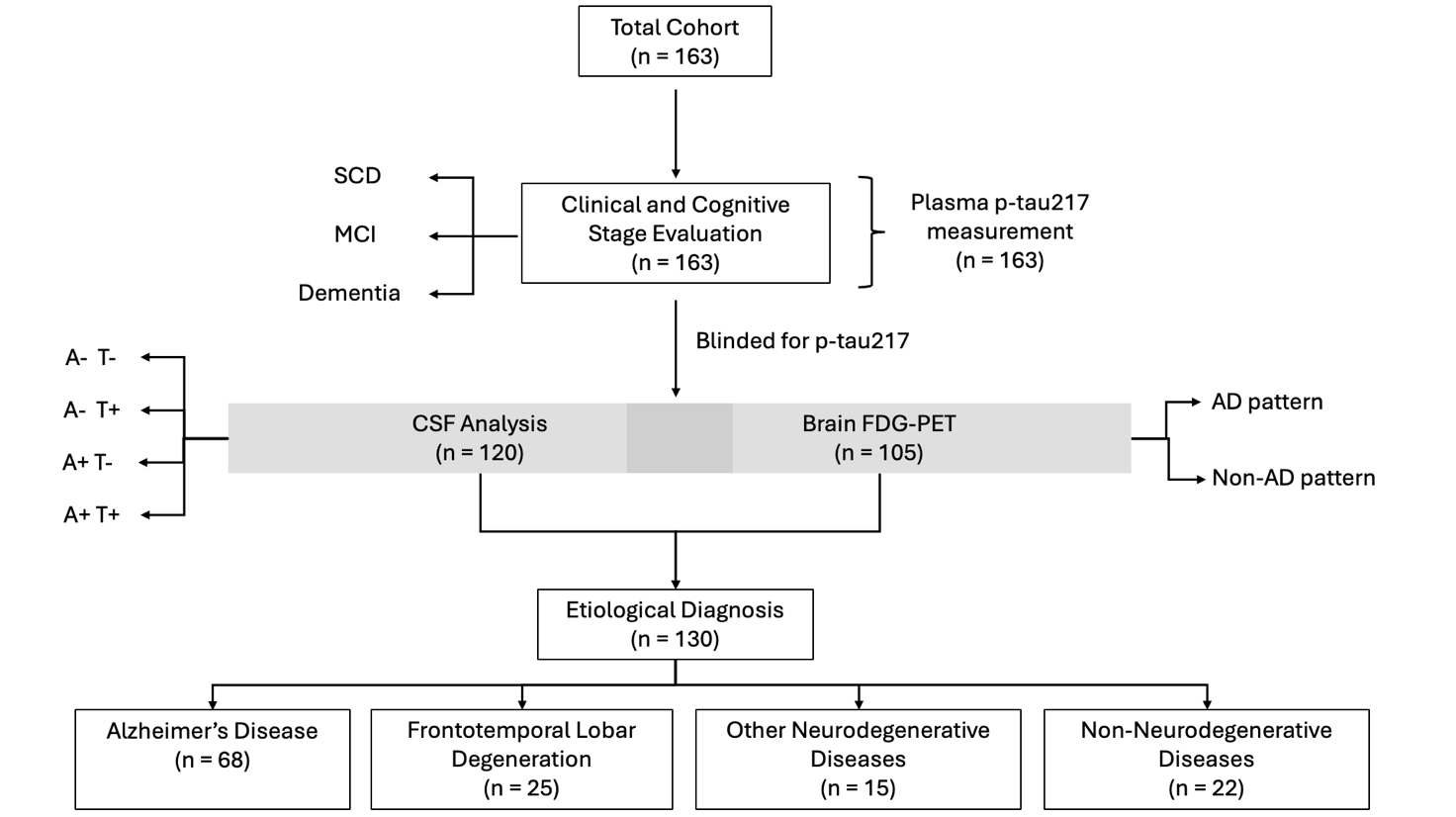
**

**Notes.** Abbreviations. SCD: subjective cognitive decline, MCI: mild cognitive impairment, CSF: cerebrospinal fluid, FDG-PET: positron emission tomography (PET) imaging with [^18^F]-fluorodeoxyglucose, AD: Alzheimer’s disease.

**Figure S2.** Boxplots of plasma p-tau217 across cognitive groups, biological and etiological classification


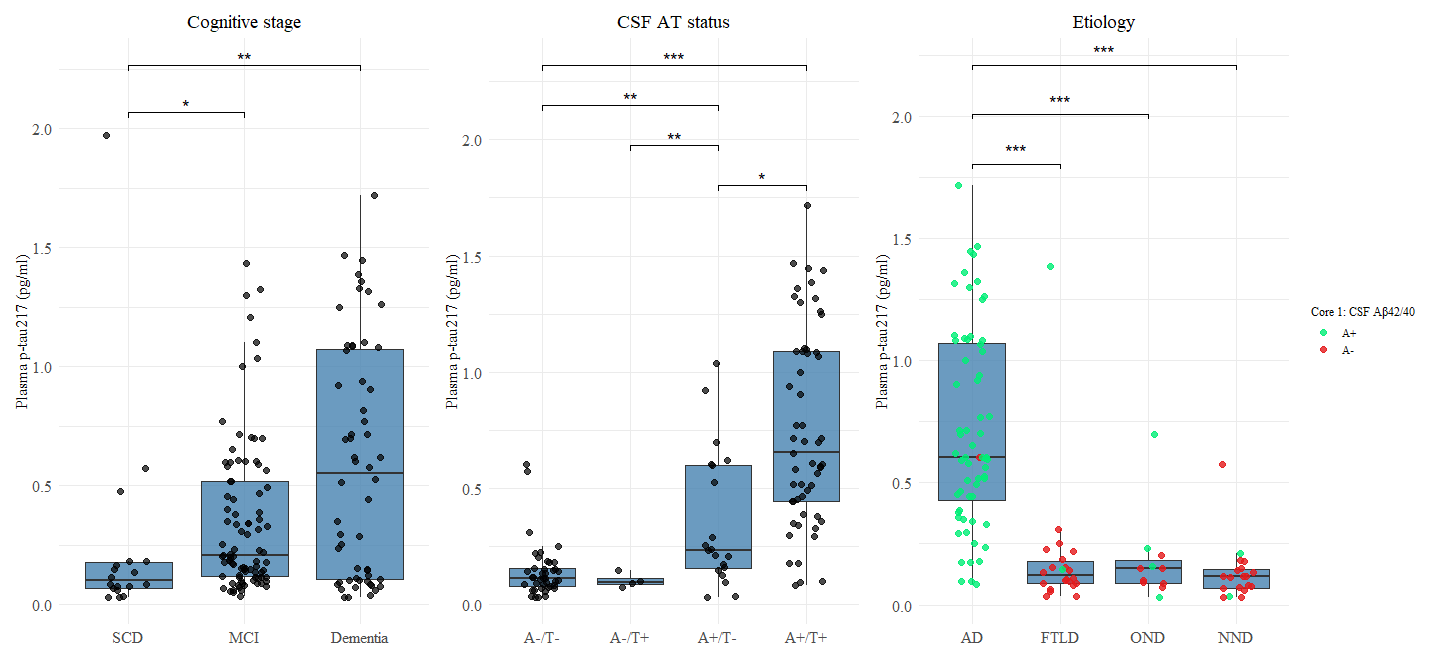


**Notes.** Boxplots illustrate key differences in p-tau217 across cognitive stages (A), CSF A/T status (B) and etiological classification (C).

*Abbreviations*. SCD: subjective cognitive decline, MCI: mild cognitive impairment, CSF: cerebrospinal fluid, AD: Alzheimer’s disease, FTLD: frontotemporal lobar degeneration, OND: other neurodegenerative disorders, NND: non-neurodegenerative disorder.

Statistical significance is reported as *** p < 0.001; ** p < 0.01; * p < 0.05.

**Figure S3.** Optimal cut-off approach of p-tau217 for CSF A- vs A+

**
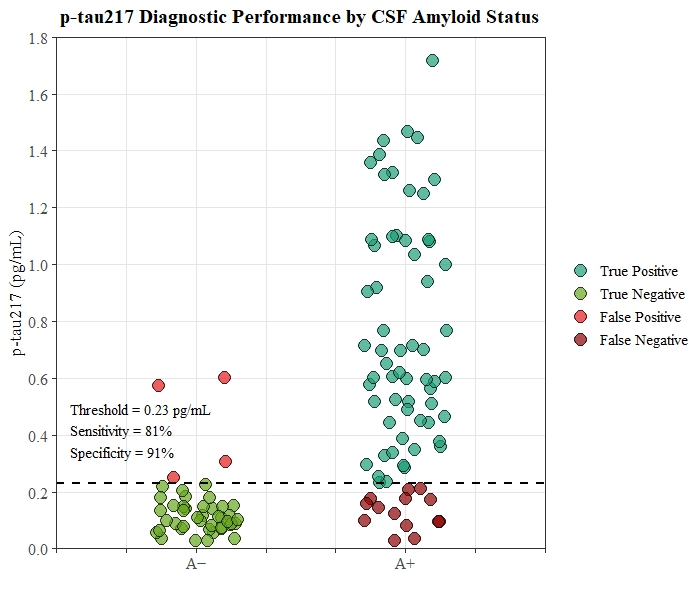
**

**Notes.** Scatterplot showing plasma p-tau217 concentrations according to CSF amyloid status (A–/A+).

**Figure S4.** CSF biomarkers profile of patients in the intermediate plasma p-tau217 range.

**
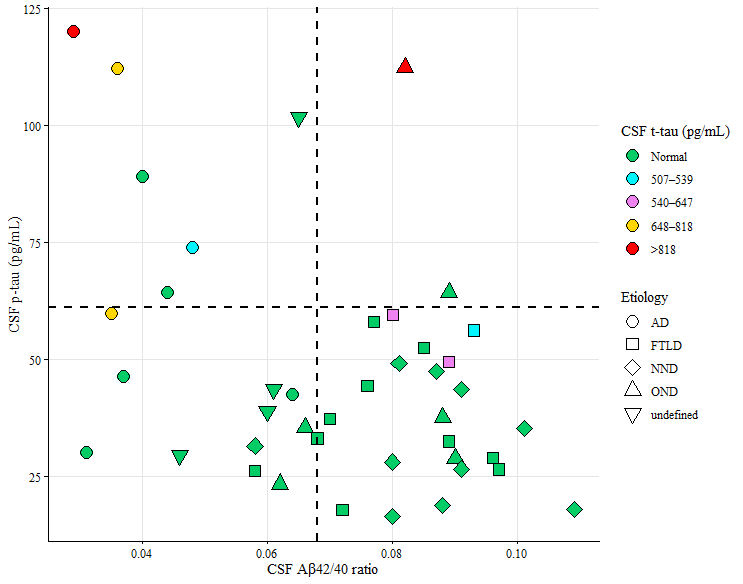
**

**Notes.** This scatter plot represents the distribution of individuals with intermediate plasma p-tau217 value among core CSF biomarkers. Dotted lines indicate thresholds for CSF p-tau (61 pg/mL) and CSF Aβ42/40 ratio (0.068). CSF t-tau values are represented with ranges by colors.

**Figure S5.** Representative patient cases illustrating multimodal diagnostic integration in our cohort.


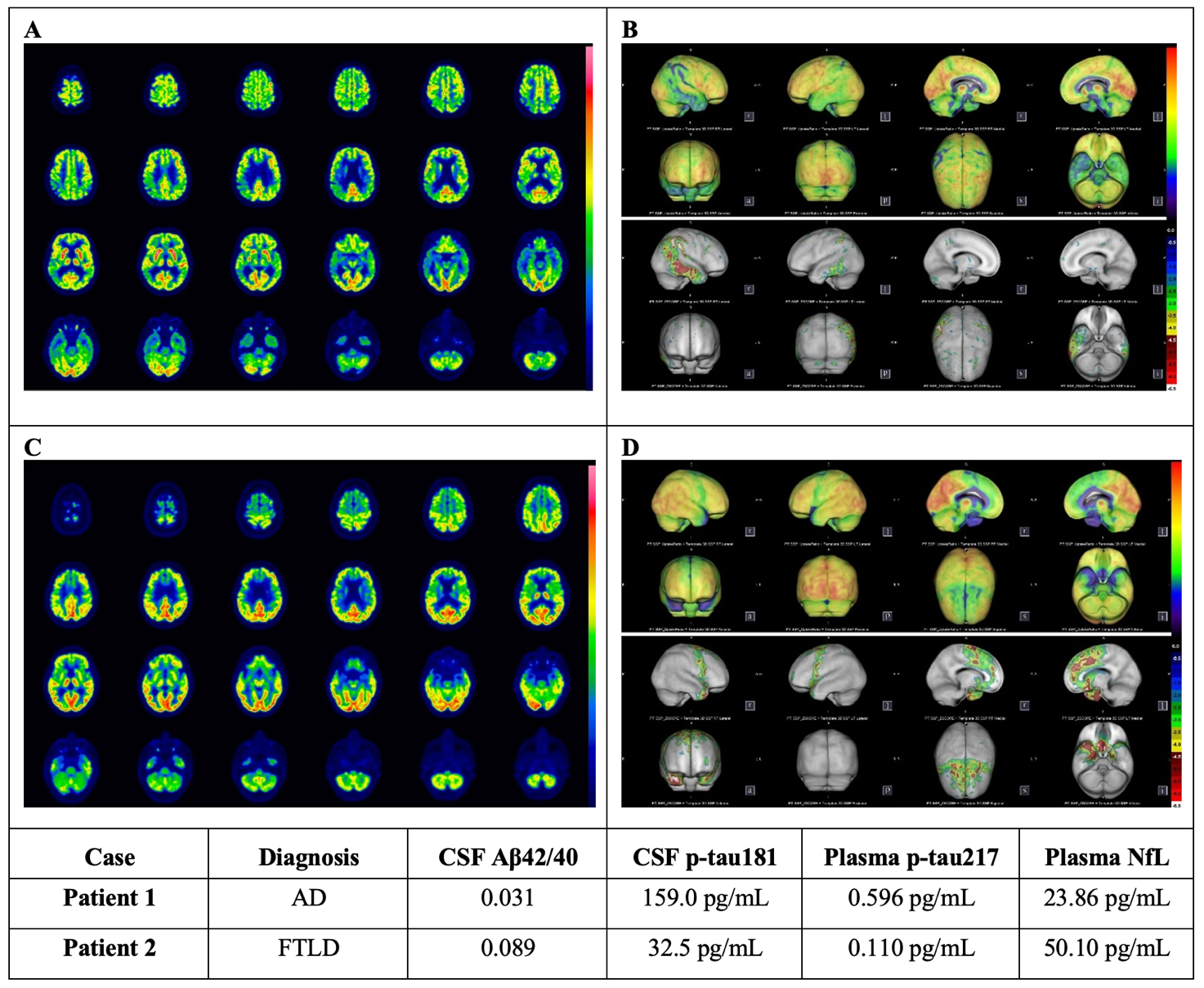


**Notes.** Representative cases from our cohort with a diagnosis of AD (Patient 1) and FTLD (Patient 2). Panels A–D show brain FDG-PET scans and QBrain renderings. Patient 1 (Panels A and B) displayed the classical posterior hypometabolism typical of AD, in line with a CSF A+/T+ profile, elevated plasma p-tau217 (0.596 pg/mL), and lower plasma NfL (23.86 pg/mL). Patient 2 (Panels C and D) showed predominant anterior hypometabolism consistent with FTLD, with a CSF A−/T− profile, lower plasma p-tau217 (0.110 pg/mL), and higher plasma NfL (50.10 pg/mL). Together, these cases exemplify the complementary role of fluid biomarkers and FDG-PET in supporting differential diagnosis within a real-world multimodal framework

*Abbreviations*. AD: Alzheimer’s disease, FTLD: frontotemporal lobar degeneration, CSF: cerebrospinal fluid., NfL: neurofilament light chain.

**Figure S6.** Exploratory plasma NfL cut-off in plasma p-tau217 negative individuals.

**
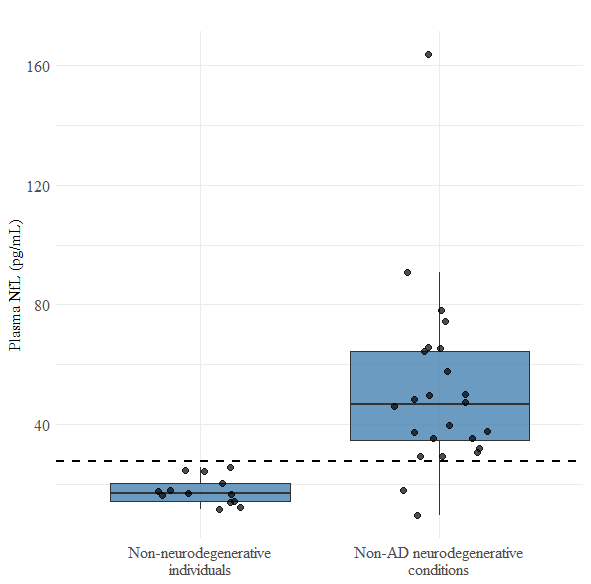
**

**Notes.** Exploratory plasma NfL cut-off for non-AD neurodegenerative diseases in p-tau217–negative individuals. Boxplots show plasma NfL levels in non-neurodegenerative and non-AD neurodegenerative conditions (OND and FTLD), after exclusion of AD cases and individuals with undefined etiology. The dashed line indicates the exploratory NfL threshold yielding 100% specificity and 92% sensitivity (27.5 pg/mL).

**Table S1.** Performance metrics at rule-out and rule-in double cut-off values derived from ROC analysis.

| **Strategy** | **Threshold** | **Sensitivity** | **Specificity** | **PPV** | **NPV** | **Accuracy** |
| --- | --- | --- | --- | --- | --- | --- |
| **Rule-out** | 0.096 pg/mL | 95% | 40% | 96% | 86% | 94% |
| **Rule-in** | 0.319 pg/mL | 73% | 95% |  |  |  |

**Notes.** PPV: positive predictive value. NPV: negative predictive value.

**Table S2.** Performance metrics at rule-out and rule-in double cut-off values derived from Palmqvist et al. (2025)

| **Strategy** | **Threshold** | **Sensitivity** | **Specificity** | **PPV** | **NPV** | **Accuracy** |
| --- | --- | --- | --- | --- | --- | --- |
| **Rule-out** | 0.220 pg/mL | 81% | 88% | 96% | 74% | 85% |
| **Rule-in** | 0.340 pg/mL | 71% | 96% |  |  |  |

**Notes.** PPV: positive predictive value. NPV: negative predictive value.

**Table S3.** Demographics, etiology distribution and biomarker status of intermediate plasma p-tau217 value subjects across CSF core 1 biomarker.

| **Variable** | **Overall**  **(n = 42)** | **A –**  **(n = 25)** | **A +**  **(n = 17)** | **p-value** |
| --- | --- | --- | --- | --- |
| **Demographic** | | | | |
| Age (years) | 42 | 70.21 (7.4) | 71.44 (6.10) | 0.6 |
| Gender, female | 42 | 10 (40%) | 12 (71%) | 0.1 |
| **Etiology** | | | | |
| Alzheimer’s disease | 9 | 0 (0%) | 9 (53%) |  |
| Frontotemporal lobar degeneration | 13 | 12 (48%) | 1 (5.9%) |  |
| Other neurodegenerative diseases | 6 | 4 (16%) | 2 (12%) |  |
| Non-neurodegenerative diseases | 10 | 9 (36%) | 1 (5.9%) |  |
| **Biomarkers** | | | | |
| CSF Aβ42/40 | 42 | 0.09 (0.01) | 0.05 (0.01) | **< 0.001** |
| CSF p-tau181 (pg/mL) | 42 | 40.90 (20.47) | 56.95 (31.51) | 0.13 |
| Plasma p-tau217 (pg/mL) | 42 | 0.16 (0.05) | 0.19 (0.07) | 0.12 |
| Plasma p-tau181 (pg/mL) | 30 | 1.25 (0.93) | 1.23 (0.37) | 0.2 |
| Plasma NfL (pg/mL) | 18 | 38.52 (18.05) | 24.66 (8.38) | 0.085 |

**Notes**. Numerical data are presented as the mean (standard deviation), while categorical data are expressed as the number (percentage of the total).

**STARD (Standards for Reporting Diagnostic accuracy studies) Checklist**

|  | **Section & Topic** | **No** | **Item** | **Reported on page #** |
| --- | --- | --- | --- | --- |
|  |  |  |  |  |
|  | **TITLE OR ABSTRACT** |  |  |  |
|  |  | **1** | Identification as a study of diagnostic accuracy using at least one measure of accuracy  (such as sensitivity, specificity, predictive values, or AUC) | 2 |
|  | **ABSTRACT** |  |  |  |
|  |  | **2** | Structured summary of study design, methods, results, and conclusions  (for specific guidance, see STARD for Abstracts) | 2 |
|  | **INTRODUCTION** |  |  |  |
|  |  | **3** | Scientific and clinical background, including the intended use and clinical role of the index test | 3 |
|  |  | **4** | Study objectives and hypotheses | 3 |
|  | **METHODS** |  |  |  |
|  | *Study design* | **5** | Whether data collection was planned before the index test and reference standard  were performed (prospective study) or after (retrospective study) | 4-5 |
|  | *Participants* | **6** | Eligibility criteria | 5 |
|  |  | **7** | On what basis potentially eligible participants were identified  (such as symptoms, results from previous tests, inclusion in registry) | 5 |
|  |  | **8** | Where and when potentially eligible participants were identified (setting, location and dates) | 4-5 |
|  |  | **9** | Whether participants formed a consecutive, random or convenience series | 4 |
|  | *Test methods* | **10a** | Index test, in sufficient detail to allow replication | 5-6 |
|  |  | **10b** | Reference standard, in sufficient detail to allow replication | 5-6 |
|  |  | **11** | Rationale for choosing the reference standard (if alternatives exist) | 5-6 |
|  |  | **12a** | Definition of and rationale for test positivity cut-offs or result categories  of the index test, distinguishing pre-specified from exploratory | 5-6 |
|  |  | **12b** | Definition of and rationale for test positivity cut-offs or result categories  of the reference standard, distinguishing pre-specified from exploratory | 5-6 |
|  |  | **13a** | Whether clinical information and reference standard results were available  to the performers/readers of the index test | 6 |
|  |  | **13b** | Whether clinical information and index test results were available  to the assessors of the reference standard | 6 |
|  | *Analysis* | **14** | Methods for estimating or comparing measures of diagnostic accuracy | 7 |
|  |  | **15** | How indeterminate index test or reference standard results were handled | 4-6 |
|  |  | **16** | How missing data on the index test and reference standard were handled | 6 |
|  |  | **17** | Any analyses of variability in diagnostic accuracy, distinguishing pre-specified from exploratory | 7 |
|  |  | **18** | Intended sample size and how it was determined | 4 |
|  | **RESULTS** |  |  |  |
|  | *Participants* | **19** | Flow of participants, using a diagram | 7 |
|  |  | **20** | Baseline demographic and clinical characteristics of participants | 7-8 |
|  |  | **21a** | Distribution of severity of disease in those with the target condition | 8-9 |
|  |  | **21b** | Distribution of alternative diagnoses in those without the target condition | 7-9 |
|  |  | **22** | Time interval and any clinical interventions between index test and reference standard | 5 |
|  | *Test results* | **23** | Cross tabulation of the index test results (or their distribution)  by the results of the reference standard | 7-9 |
|  |  | **24** | Estimates of diagnostic accuracy and their precision (such as 95% confidence intervals) | 7-9 |
|  |  | **25** | Any adverse events from performing the index test or the reference standard | N.A. |
|  | **DISCUSSION** |  |  |  |
|  |  | **26** | Study limitations, including sources of potential bias, statistical uncertainty, and generalisability | 10-12 |
|  |  | **27** | Implications for practice, including the intended use and clinical role of the index test | 10-12 |
|  | **OTHER INFORMATION** |  |  |  |
|  |  | **28** | Registration number and name of registry | N.A. |
|  |  | **29** | Where the full study protocol can be accessed | N.A. |
|  |  | **30** | Sources of funding and other support; role of funders | 13 |
|  |  |  |  |  |
